# Regulation of Surface Structure of [Au_9_Ag_12_(SAdm)_4_(Dppm)_6_Cl_6_](SbF_6_)_3_ Nanocluster *via* Alloying

**DOI:** 10.3389/fchem.2021.793339

**Published:** 2022-01-24

**Authors:** Huijuan Deng, Xiaowu Li, Xiaoxun Yan, Shan Jin, Manzhou Zhu

**Affiliations:** ^1^ Department of Chemistry and Centre for Atomic Engineering of Advanced Materials, Key Laboratory of Structure and Functional Regulation of Hybrid Materials of Ministry of Education, Anhui University, Hefei, China; ^2^ Anhui Province Key Laboratory of Chemistry for Inorganic/Organic Hybrid Functionalized Materials, Institutes of Physical Science and Information Technology, Anhui University, Hefei, China

**Keywords:** regulation of surface structure, alloy engineering, optical properties, electrochemical properties, intercluster reactions

## Abstract

Tailoring of specific sites on the nanocluster surface can tailor the properties of nanoclusters at the atomic level, for the in-depth understanding of structure and property relationship. In this work, we explore the regulation of surface structure of [Au_9_Ag_12_(SAdm)_4_(Dppm)_6_Cl_6_](SbF_6_)_3_ nanocluster *via* alloying. We successfully obtained the well-determined tri-metal [Au_9_Ag_8_@Cu_4_(SAdm)_4_(Dppm)_6_Cl_6_](SbF_6_)_3_ by the reaction of [Au_9_Ag_12_(SAdm)_4_(Dppm)_6_Cl_6_](SbF_6_)_3_ with the Cu^I^(SAdm) complex precursor. X-ray crystallography identifies that the Cu dopants prioritily replace the position of the silver capped by Dppm ligand in the motif. The Cu doping has affected the optical properties of Au_9_Ag_12_ alloy nanocluster. DPV spectra, CD spectra and stability tests suggest that the regulation of surface structure *via* Cu alloying changes the electronic structure, thereby affecting the electrochemical properties, which provides insight into the regulation of surface structure of [Au_9_Ag_12_(SAdm)_4_(Dppm)_6_Cl_6_](SbF_6_)_3_ *via* alloying.

## Introduction

Atomically precise core-shell nanoclusters have become a promising material in catalysis, biomedicine, and chemical sensing due to the unique quantum confinement effect resulting in optical properties (Jin et al., 2016; Yao et al., 2018; Xu et al., 2019; Jin R. et al., 2021; Sun et al., 2021; Zheng et al., 2021). The studies on correlation between the properties and structures of cluster compounds based on the determined crystal structures show that the core and shell structures have different effects on the performance of the cluster compounds, and modifications on the core and shell structures may induce variations on clusters properties (AbdulHalim et al., 2014; Chakraborty and Pradeep, 2017; Khatun et al., 2018; Yan et al., 2018; Jin Y. et al., 2021). The Pt core-doped nanocluster PtAu_24_(SC_6_H_13_)_18_ exhibits higher hydrogen production than that of Au_25_ (Kwak et al., 2017), and the dopant AuAg_24_ shows stronger fluorescence performance (Bootharaju et al., 2016). Surface shell dopant Au_24_Cu_6_ exhibited superior catalytic activity compared to other homometallic and Au-Cu alloy nanoclusters (Chai at al., 2019). Therefore, alloying could serve as an efficient approach to tailor the properties of nanoclusters for more applications (Ghosh et al., 2018; Jin et al., 2018a; Wang et al., 2018; Dias and Leite, 2019).

Current alloy research mainly focuses on bimetallic clusters, and there are few studies on trimetallic clusters due to factors such as synthesis, characterization, and crystallization, etc. (Kang et al., 2016; Sharma et al., 2016; Yan et al., 2016; Hossain, et al., 2018; Kang et al., 2019a; Kang et al., 2019b; Kang et al., 2020) When the third metal is doped into the bimetallic alloy clusters, what site will it occupy and what effect will it have on the overall performance?Recently, for the active metal Cu doping, several surface Cu-doped nanoclusters such as Au_13_Cu_x_ (x = 2, 4, 8) (Yang et al., 2013), Cu_x_Au_25-x_ (Yang et al., 2017), Cu_3_Au_34_ (Yang et al., 2017), Ag_28_Cu_12_ (Yan et al., 2016), Ag_30_Cu_14_ (Li at al., 2020) and Cu-internal-doped nanoclusters like Ag_61_Cu_30_ have been observed and well-determined by x-ray crystallography (Zou et al., 2020). Specifically, the outer Au shells always are partially alloyed by the incorporated Cu heteroatoms for Au-based nanoclusters, while core-shell alloy nanoclusters with a shell-by-shell configuration could be generated for Ag-based nanoclusters. However, for the Au-Ag alloy nanocluster, how will the copper atoms choose the sites?

**SCHEME 1:**
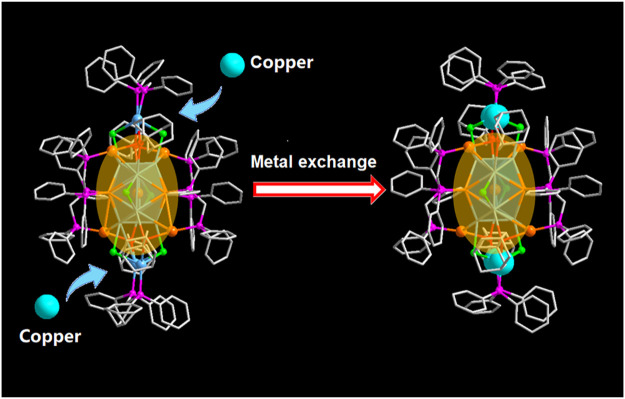
The metal exchange from [Au9Ag12(SAdm)4(Dppm)6Cl6](SbF6)3 to [Au9Ag8Cu4(SAdm)4(Dppm)6Cl6](SbF6)3 (Color labels: Golden = Au; Sky blue = Ag; red = S; purple = P; Gray = C; light green = Cl; Turquoise = Copper).

Herein, we use position-determined alloy clusters [Au_9_Ag_12_(SAdm)_4_(Dppm)_6_Cl_6_](SbF_6_)_3_ as templates for the doping of the third metal copper (Jin et al., 2018b). The crystallography analysis suggested that the four Cu atoms priority replace the position of the silver capped by Dppm ligand in the motif for [Au_9_Ag_8_Cu_4_(SAdm)_4_(Dppm)_6_Cl_6_](SbF_6_)_3_ (Scheme 1). And the Cu doping affected the electronicstructure, resulting in the difference of optical properties in CD spectra, DPV spectra and so on. This provides a good observation method for understanding the doping position.

## Materials and Methods

### Materials

Tetrachloroauric(III) acid (HAuCl4.3H_2_O, 99.99%), silver nitrate (AgNO_3_, 98%), tetrabutyl ammonium chloride (TBAC, 98%), sodium borohydride (NaBH_4_, 99.99%), bis-(diphenylphosphino)methane (Dppm, 98%), 1-Adamantanethiol (C_10_H_16_S, 99%), sodium hexafluoroantimonate (NaSbF_6_, 98%), toluene (Tol, HPLC grade, Aldrich), methanol (CH_3_OH, HPLC, Aldrich), n-hexane (Hex, HPLC grade, Aldrich), dichloromethane (CH_2_Cl_2_, HPLC grade, Aldrich), Pure water was purchased from Wahaha Co. Ltd. All reagents were used as received without further purification.

### Synthesis of [Au_9_Ag_12_(SAdm)_4_(Dppm)_6_Cl_6_] (SbF_6_)_3_ nanocluster

The synthesis of [Au_9_Ag_12_(SAdm)_4_(Dppm)_6_Cl_6_] was obtained by the method reported (Jin et al., 2018a). Typically, HAuCl_4_ 3H_2_O (40 mg) and AgNO_3_ (60 mg) was mixed in 15 ml toluene with TBAC (200 mg). Stirring for 5min, 50 mg bis-(diphenylphosphino)methane and 50 mg 1-Adamantanethiol were added together. 15 min later, a solution of 20 mg NaBH_4_ (1 ml H_2_O) was added. The reaction sustained for 12 h at room temperature. The crude product was spied dry and washed by hexane. 30 mg NaSbF_6_ in 3 ml CH_3_OH was added to replace the anion of the cluster for easy crystallization. The yield of [Au_9_Ag_12_(SAdm)_4_(Dppm)_6_Cl_6_](SbF_6_)_3_ is as high as 70% based on the Ag element, which was determined by ESI-MS and X-ray crystallography. The CCDC number is 2114779.

### Synthesis of Cu^I^SR Complex Precursor

CuCl (0.05 g, 0.5 mmol) was dissolved in 5 ml CH_3_CN, and AdmSH (0.09 g, 0.55 mmol) was dissolved in 5 ml CH_3_CN and added drop-wise to the solution under vigorously stirred. The resulting solution mixture was then washed several times with hexane. Then the final product was used directly.

### Synthesis of [Au_9_Ag_8_Cu_4_(SAdm)_4_(Dppm)_6_Cl_6_](SbF_6_)_3_ nanocluster:

The 20 mg [Au_9_Ag_12_(SAdm)_4_(Dppm)_6_Cl_6_](SbF_6_)_3_ dissolved in 7 ml methylene chloride, Cu^I^SR (1 mg) was added to the solution. The reaction lasted for 10 min at room temperature. After that, the reaction mixture was centrifuged at 8,000 rpm. The organic layer was separated from the precipitate and evaporated to dryness. [Au_9_Ag_8_Cu_4_(SAdm)_4_(Dppm)_6_Cl_6_](SbF_6_)_3_ was obtained. The yield of [Au_9_Ag_8_Cu_4_(SAdm)_4_(Dppm)_6_Cl_6_](SbF_6_)_3_ is as high as 60% based on the [Au_9_Ag_12_(SAdm)_4_(Dppm)_6_Cl_6_](SbF_6_)_3._ Orange crystals were crystallized from CH_2_Cl_2_/hexane at room temperature after 7 days. The CCDC number is 2114780.

### Characterization

All UV/Vis absorption spectra of nanoclusters are recorded on a Techcomp UV1000 spectrophotometer. Electrospray ionization time-of-flight mass spectrometry (ESI-TOF-MS) measurement was performed using a UPLC H-class/XEV0G2-XS QTOF high-resolution mass spectrometer. The sample was directly infused into the chamber at 5 μL/min. Photoluminescence spectra were measured using an FL-7000 spectrofluorometer with the same optical density (OD) of ∼0.2. X-ray photoelectron spectroscopy (XPS) measurements were performed using a Thermo ESCALAB 250 configured with a monochromated Al Kα (1486.8 eV) 150 W X-ray source, 0.5 m mm circular spot size, a flood gun to counter charging effects, and an analysis chamber base pressure lower than 1 × 10^–9^ mbar, and the data were collected with FAT = 20 eV. CD spectra are recorded with a BioLogic MOS-500 CD-spectropolarimeter in a 0.1-cm path length quartz cell. The spectra are recorded in diluted solutions of dichloromethane and the signal of the blank solvent is subtracted. The enantiomers of chiral [Au_9_Ag_8_Cu_4_(SAdm)_4_(Dppm)_6_Cl_6_](SbF_6_)_3_ were separated by HPLC on an Agilent 1260 system equipped with a Chiralcel OD-H column (5 µm, 4.6 mm ø × 250 mm). A diode array detector (DAD) *in situ* monitors the entire optical absorption spectrum (190–950 nm range) of the eluted solution, and the 427, 482 and 710 nm wavelength were used for the chromatogram. The nanoclusters were pre-dissolved in solvent which has the same composition of the mobile phase (methanol/isopropanol = 35/65). The flow rate was at 0.4 ml min^−1^ and the temperature set at 20°C.

## Results and Discussion

**FIGURE 1:**
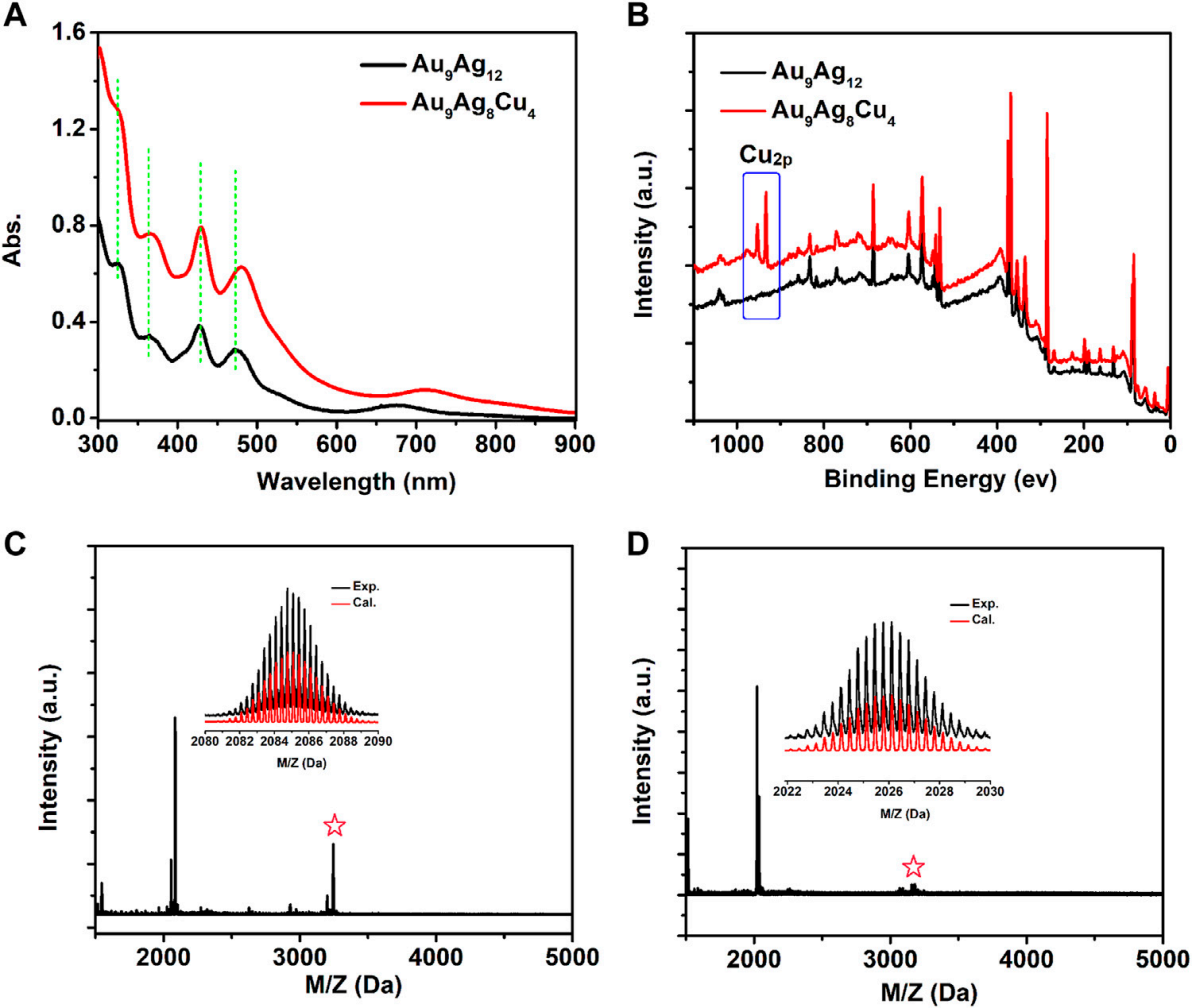
**(A)** Optical absorption spectra and **(B)** the XPS spectra of [Au_9_Ag_12_(SAdm)_4_(Dppm)_6_Cl_6_](SbF_6_)_3_ (black line) and [Au_9_Ag_8_Cu_4_(SAdm)_4_(Dppm)_6_Cl_6_](SbF_6_)_3_ (red line); ESI-MS spectra of **(C)** [Au_9_Ag_12_(SAdm)_4_(Dppm)_6_Cl_6_](SbF_6_)_3_ and **(D)** [Au_9_Ag_8_Cu_4_(SAdm)_4_(Dppm)_6_Cl_6_](SbF_6_)_3._ The peaks labeled by asterisks in Panels **(C, D)** correspond to [Au_9_Ag_12_(SAdm)_4_(Dppm)_6_Cl_6_+(SbF_6_)]^2+^ and [Au_9_Ag_8_Cu_4_(SAdm)_4_(Dppm)_6_Cl_6_+SbF_6_)^2+^, respectively.

The synthesized [Au_9_Ag_12_(SAdm)_4_(Dppm)_6_Cl_6_](SbF_6_)_3_ based on the reported method was determined by ESI-MS and X-ray crystallography. The next is the regulation of surface structure of [Au_9_Ag_12_(SAdm)_4_(Dppm)_6_Cl_6_](SbF_6_)_3_ with Cu^I^(SAdm) complex precursor. As shown in Figure 1A, the [Au_9_Ag_12_(SAdm)_4_(Dppm)_6_Cl_6_]^3+^ shows main peaks at 322, 365, 427, 480 and 670 nm, respectively, and [Au_9_Ag_8_Cu_4_(SAdm)_4_(Dppm)_6_Cl_6_]^3+^ shows 322, 366, 427, 482 and 710 nm, respectively. In contrast, most of the peaks for both nanoclusters have not changed significantly, except for the red shift of the 670 nm peak to 710 nm. The binding energy of Cu_2p_ from XPS data confirmed the Cu doping in the [Au_9_Ag_8_Cu_4_(SAdm)_4_(Dppm)_6_Cl_6_]^3+^ (Figure 1B), and energy level positions of other elements have basically not changed (Supplementary Figure S1). The peak at m/z 2084.85 corresponds to the 3 + charge of [Au_9_Ag_12_(SAdm)_4_(Dppm)_6_Cl_6_] and can be perfectly assigned by the calculated result (m/z 2084.81) (Figure 1C). And peak at m/z 2026.20 corresponds to the 3 + charge of [Au_9_Ag_8_Cu_4_(SAdm)_4_(Dppm)_6_Cl_6_] (Cal. 2026.18) (Figure 1D). Meanwhile, the H-NMR and ^1^H–^1^H COSY spectra of [Au_9_Ag_12_(SAdm)_4_(Dppm)_6_Cl_6_](SbF_6_)_3_ and Au_9_Ag_8_Cu_4_(SAdm)_4_(Dppm)_6_Cl_6_](SbF_6_)_3_ nanoclusters were performed, showing that the overall chemical environment is weakly affected by copper doping regulation (Supplementary Figures S2, 3).

**FIGURE 2:**
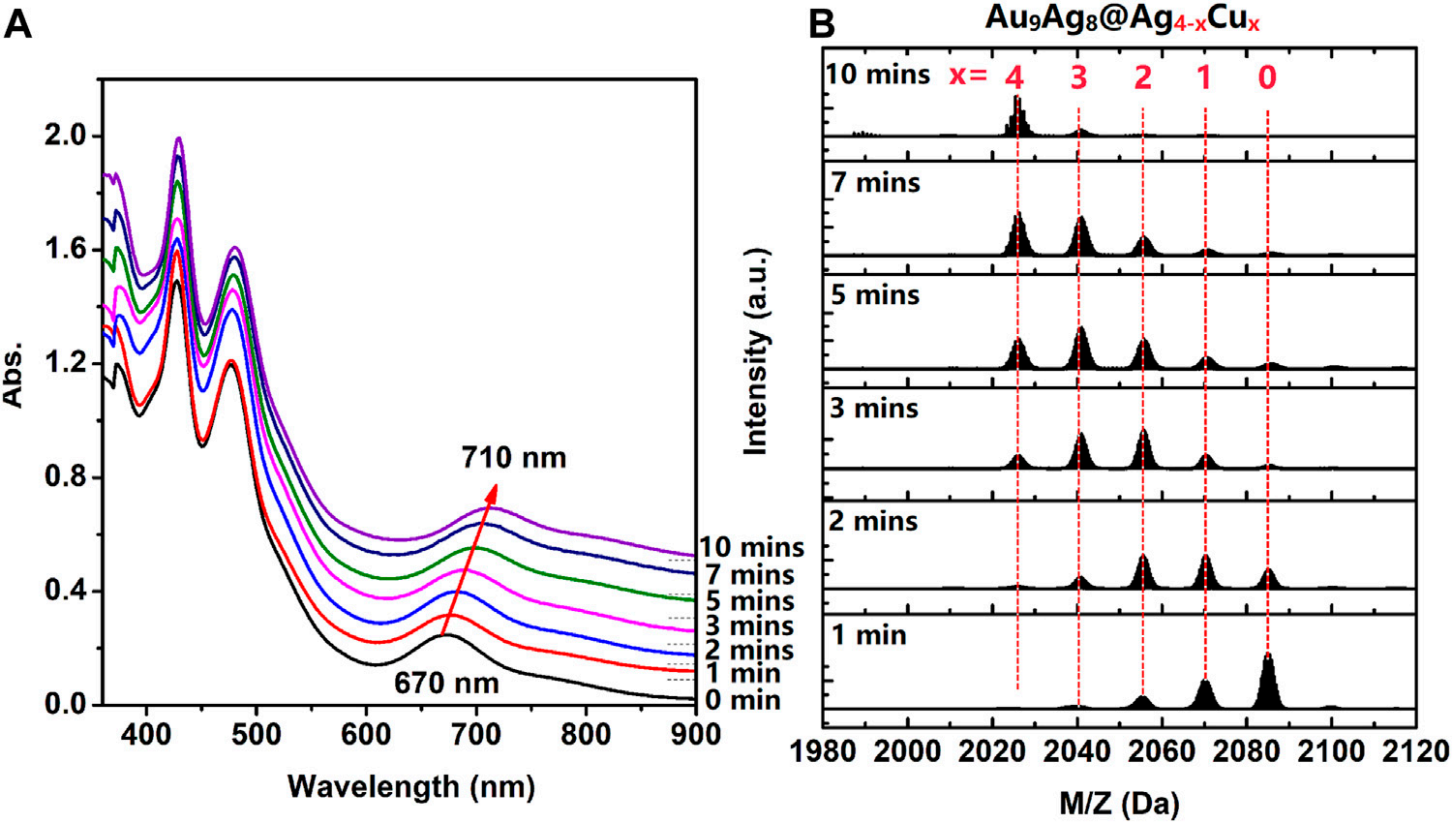
**(A)** the time-dependent UV-Vis spectra and **(B)** ESI mass spectra of [Au_9_Ag_12_(SAdm)_4_(Dppm)_6_Cl_6_](SbF_6_)_3_ in CH_2_Cl_2_ after adding Cu^I^(SAdm) complex precursor.

Furthermore, in order to have a deep understanding of the regulation process, the time-dependent UV-Vis spectra and ESI mass spectra of [Au_9_Ag_12_(SAdm)_4_(Dppm)_6_Cl_6_](SbF_6_)_3_ in CH_2_Cl_2_ after adding Cu^I^(SAdm) complex precursor were performed. As shown in Figure 2A, with the increase of time of Cu^I^(SAdm) complex precursor. adding, the peak centered at 427 nm always maintained, and the peak centered at 480 only 2 nm redshifts. While the 670 nm peak gradually red shift to 710 nm, with a redshift value of 40 nm. ESI mass spectra suggested the copper atoms are gradually replacing silver atoms, which leads to red shift (Figure 2B). The successful determination of [Au_9_Ag_8_Cu_4_(SAdm)_4_(Dppm)_6_Cl_6_]^3+^ structure allowed us to know the site of doping clearly.

**FIGURE 3:**
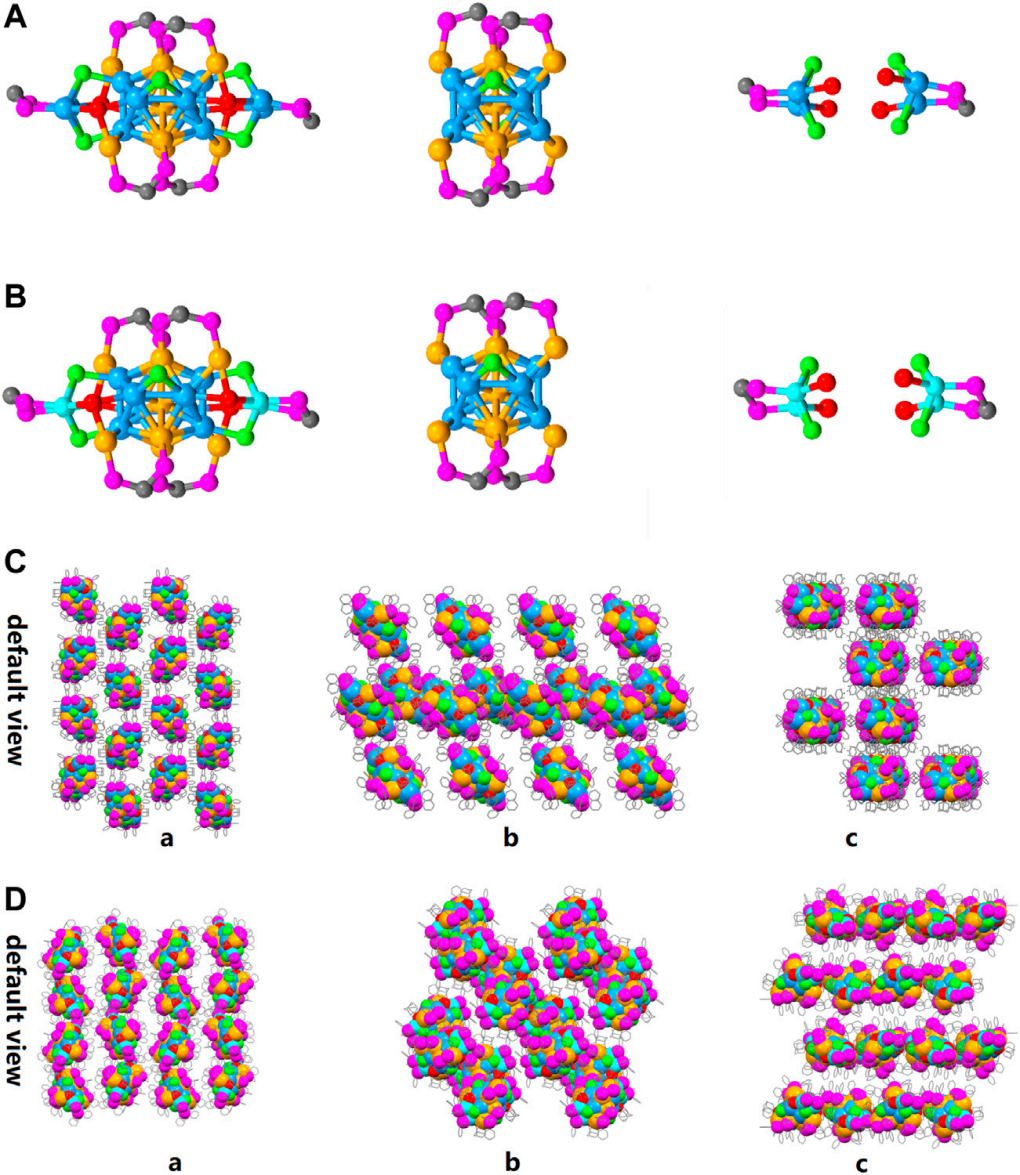
**(A)** the overall structure of [Au_9_Ag_12_(SAdm)_4_(Dppm)_6_Cl_6_](SbF_6_)_3_; **(B)** the overall structure of [Au_9_Ag_8_Cu_4_(SAdm)_4_(Dppm)_6_Cl_6_](SbF_6_)_3_; Packing models of **(C)** [Au_9_Ag_12_(SAdm)_4_(Dppm)_6_Cl_6_]^3+^ and **(D)** [Au_9_Ag_8_Cu_4_(SAdm)_4_(Dppm)_6_Cl_6_]^3+^ from default view a, b, c. Color labels: Golden = Au; Sky blue = Ag; red = S; purple = P; Gray = C; light green = Cl; Turquoise = Copper).

As shown in Figure 3, the overall structure of [Au_9_Ag_12_(SAdm)_4_(Dppm)_6_Cl_6_](SbF_6_)_3_ and [Au_9_Ag_8_Cu_4_(SAdm)_4_(Dppm)_6_Cl_6_](SbF_6_)_3_ are basically the same: firstly, five gold atoms and eight silver atoms constitute the icosahedron, then the Au_5_Ag_8_ icosahedron and four gold atoms constitute the Au_4_@Ag_8_Au_5_ metallic kernel. The Au_4_@Ag_8_Au_5_ is first capped by four Dppm ligands and two Cl ligands, forming Au_4_@Ag_8_Au_5_(Dppm)_4_Cl_2_ framework. After the Au_4_@Ag_8_Au_5_(Dppm)_4_Cl_2_ is further protected by two peripheral structures DppmAg_2_Cl_2_(SR)_2_, the Au_9_Ag_12_ was obtained. By contrast, Au_5_Ag_8_@Au_4_@Cu_4_ is obtained when four copper atoms doped the position of the silver of peripheral structures DppmAg_2_Cl_2_(SR)_2_. Meanwhile, the copper doping has little effect on the bond length and angle of the icosahedron metal core (Supplementary Figure S4). Based on the doping sites of copper atoms, we realize that the Au_4_@Ag_8_Au_5_ will be a stable metal core. In the packing model, the difference of arrangement can be observed clearly, and it is worth mentioning that the doping can affect the arrangement of clusters in the unit cell (Figures 3C,D) from a crystal engineering point of view.

**FIGURE 4:**
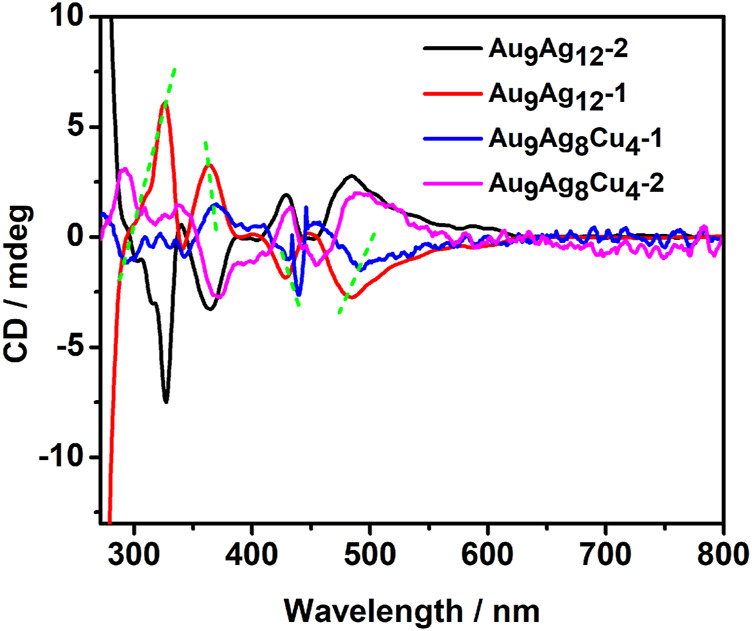
The circular dichroism (CD) spectra of the enantiomer in **(A)** [Au_9_Ag_12_(SAdm)_4_(Dppm)_6_Cl_6_](SbF_6_)_3_ and **(B)** [Au_9_Ag_8_Cu_4_(SAdm)_4_(Dppm)_6_Cl_6_](SbF_6_)_3_ nanoclusters.

As reported, the chirality of metal clusters mainly come from chiral metalcore, the arrangement of chiral ligands and local chiral patterns on an achiral surface (Zeng and Jin, 2017). The chirality of [Au_9_Ag_12_(SAdm)_4_(Dppm)_6_Cl_6_]^3+^ comes from the chiral Au_4_@Ag_8_Au_5_ metallic kernel. After doping, the cluster will have a different CD spectrum compared to the parent compound. Importantly, herein, the Cu dopants also have some impacts on the chiral properties. As shown in Figure 4, the CD spectra of [Au_9_Ag_12_(SAdm)_4_(Dppm)_6_Cl_6_]^3+^ reveal multiple CD-active peaks at 325, 363, 428 and 483 nm, respectively, and some weak peaks. While the CD spectra of [Au_9_Ag_8_Cu_4_(SAdm)_4_(Dppm)_6_Cl_6_]^3+^ shows peaks at 340, 373, 442, and 493 nm, respectively. The Au_38_ cluster with Pd atoms leads to core-doped Pd_2_Au_36_(SC_2_H_4_Ph)_24_. Comparison between the CD spectra of Au_38_(SC_2_H_4_Ph)_24_ and Pd_2_Au_36_(SC_2_H_4_Ph)_24_ shows significant differences, revealing core-doping has strong impacts on the electronic structure of the cluster (Barrabés et al., 2014). The comparison between the CD spectra of [Au_9_Ag_12_(SAdm)_4_(Dppm)_6_Cl_6_]^3+^ and [Au_9_Ag_8_Cu_4_(SAdm)_4_(Dppm)_6_Cl_6_]^3+^shows that all the peaks from [Au_9_Ag_8_Cu_4_(SAdm)_4_(Dppm)_6_Cl_6_]^3+^ have redshift, different from the differences between Au_38_(SC_2_H_4_Ph)_24_ and Pd_2_Au_36_(SC_2_H_4_Ph)_24_. The doping location may have different impacts on the CD spectra.

**FIGURE 5:**
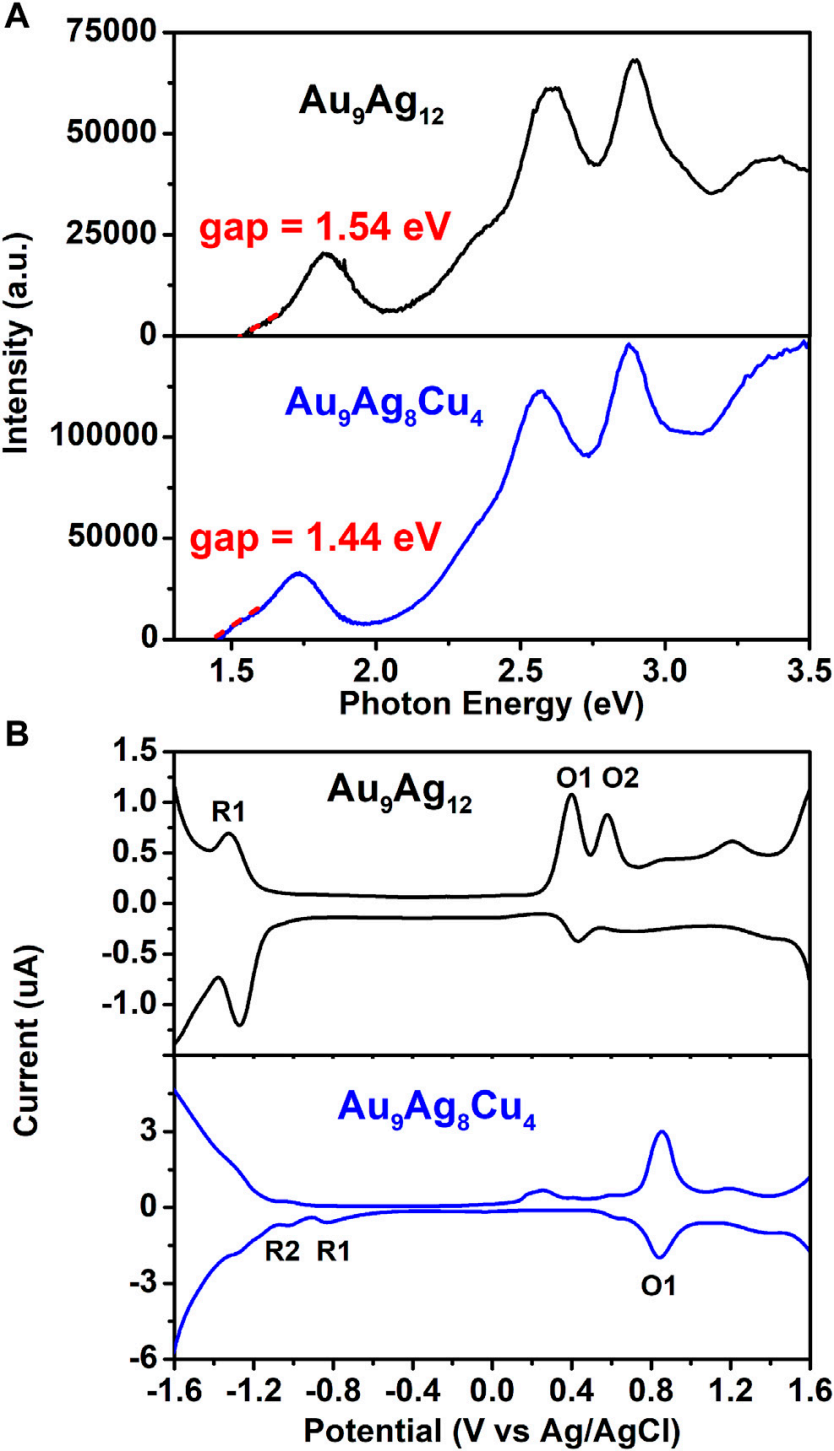
**(A)** Photon energy spectra of Au_9_Ag_12_ (black line) and Au_9_Ag_8_Cu_4_ (blue line). **(B)** DPV of Au_9_Ag_12_ (black line) and Au_9_Ag_8_Cu_4_ (blue line).

In addition to the CD spectra, the electronic structures of [Au_9_Ag_12_(SAdm)_4_(Dppm)_6_Cl_6_](SbF_6_)_3_ and [Au_9_Ag_8_Cu_4_(SAdm)_4_(Dppm)_6_Cl_6_](SbF_6_)_3_ are investigated by optical and electrochemical spectroscopies. Differential pulse voltammetry (DPV) of Au_9_Ag_12_ and Au_9_Ag_8_Cu_4_ are carried out. The scan direction was detected from +1.6 to -1.6 V and then back from −1.6 to +1.6 V. As shown in Figure 5, The HOMO-LUMO gaps of Au_9_Ag_12_ and Au_9_Ag_8_Cu_4_ are determined as 1.54 and 1.44 eV, respectively. For the differential pulse voltammetry (DPV) curves, there is a reduction peak at −1.32 V (R1) and two oxidation peaks at 0.40 V(O1) and 0.58 V (O2) for Au_9_Ag_12_, while there are two reduction peaks at −0.82 V (R1) and −1.01 (R2) and one oxidation peaks at 0.83 V (O1) for Au_9_Ag_8_Cu_4_. So, the electrochemical energy gap is 1.72 eV for Au_9_Ag_12_ and 1.65 eV for Au_9_Ag_8_Cu_4_. The HOMO-LUMO gaps calculated from DPV are consistent with those derived from the optical absorption spectra. So, the regulation of surface structure via Cu alloying changes the electronic structure, thereby affecting the electrochemical properties. Besides, the [Au_9_Ag_12_(Sadm)_4_(Dppm)_6_Cl_6_](SbF_6_)_3_ in CH_2_Cl_2_ solution shows non-fluorescence, while the Cu dopant [Au_9_Ag_8_Cu_4_(SAdm)_4_(Dppm)_6_Cl_6_](SbF_6_)_3_ in CH_2_Cl_2_ solution shows weak fluorescence at 638 nm, once again verifying the changes in the electronic structure. (Supplementary Figure S5).

**FIGURE 6:**
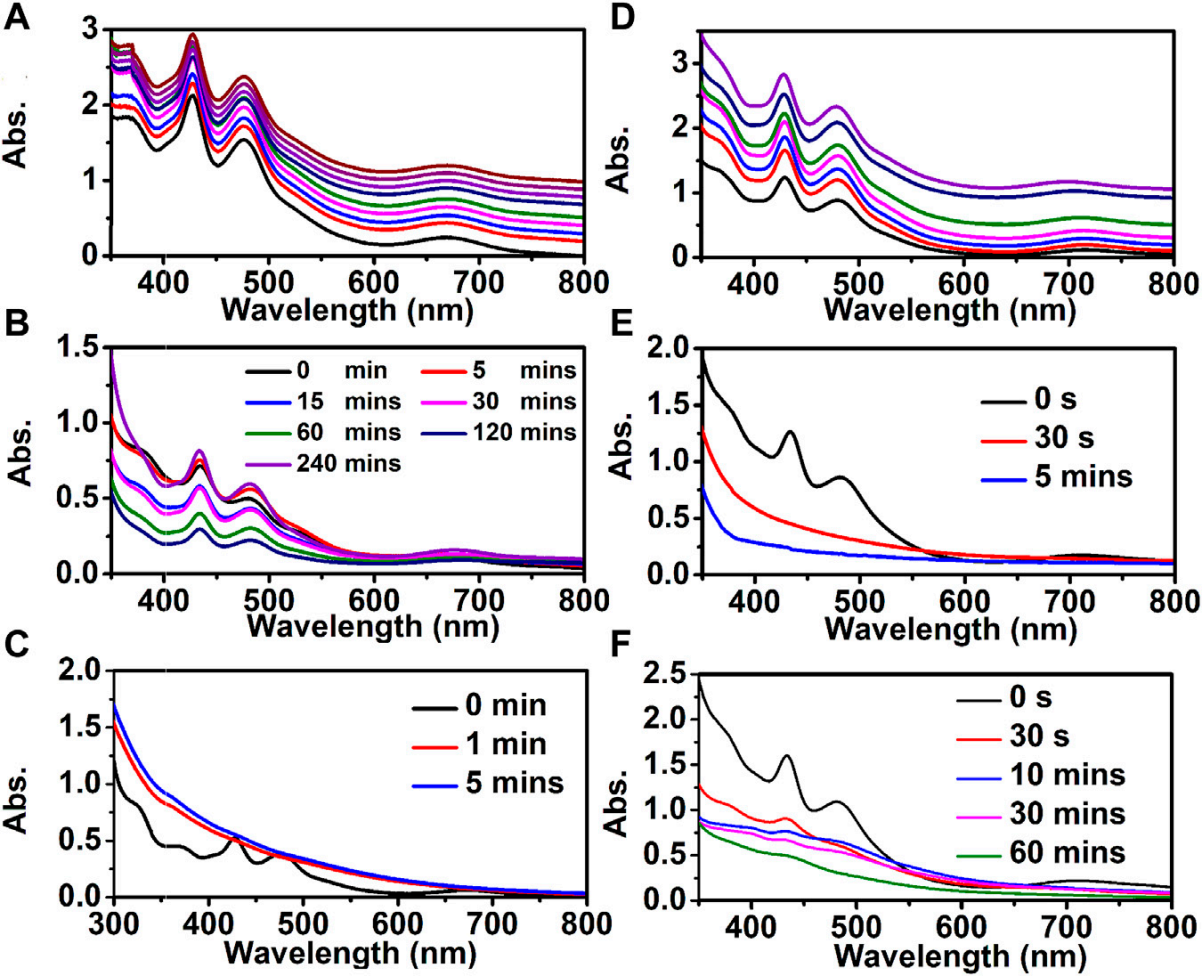
The stability test: **(A–C)** for [Au_9_Ag_12_(SAdm)_4_(Dppm)_6_Cl_6_](SbF_6_)_3_ and **(D–F)** for [Au_9_Ag_8_Cu_4_(SAdm)_4_(Dppm)_6_Cl_6_](SbF_6_)_3_ nanocluster. **(A, D)** the thermal stability test; **(B, E)** oxidizing stability test; **(C, F)** reducing stability test.

The [Au_9_Ag_12_(SAdm)_4_(Dppm)_6_Cl_6_](SbF_6_)_3_ and [Au_9_Ag_8_Cu_4_(SAdm)_4_(Dppm)_6_Cl_6_](SbF_6_)_3_ show good stability in an ambient environment (Figures 6A,D) and the stability tests (i.e., under oxidizing/reducing environments) for Au_9_Ag_12_ and Au_9_Ag_8_Cu_4_ are also performed to explore the effects of copper dopants on the stability of nanoclusters. Under the oxidizing environment (by mixing 200 μL of H_2_O_2_ (50%) with 6 mg of cluster in 10 ml of CH_2_Cl_2_), the Au_9_Ag_12_ can stabilize for several hours (Figures 6B,E), and the peaks of the UV-vis spectra are obvious. However, the Au_9_Ag_8_Cu_4_ decompose quickly to form complexes within several mins (Figures 6C,F). This difference may be because the peripheral copper atom is easier to be oxidized. Meanwhile, the copper doping has an impact on the properties of clusters on reducing environment (by mixing the 10 ml CH_2_Cl_2_ solvent of 6 mg of cluster with 200 μL of EtOH solvent of 1 mg of NaBH_4_). The UV-vis of Au_9_Ag_12_ changes quickly until there are no obvious peaks within 30s. And the UV-vis of Au_9_Ag_8_Cu_4_ also changes quickly, but still some peaks can be observed within 60 min. These indicate the regulation of surface structure affects the stability of nanoclusters.

**FIGURE 7:**
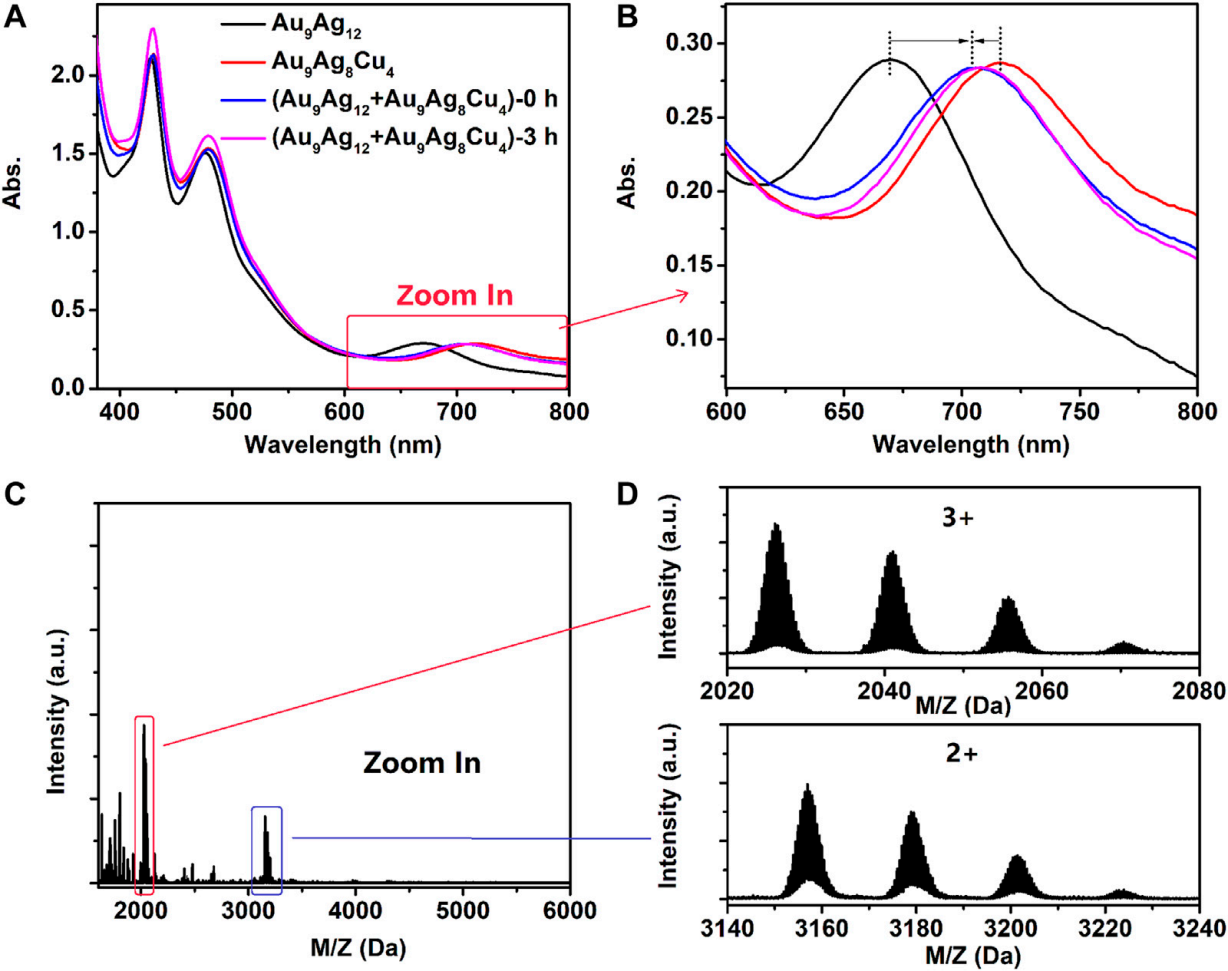
**(A, B)** the UV-vis spectra of intercluster reaction between [Au_9_Ag_12_(SAdm)_4_(Dppm)_6_Cl_6_](SbF_6_)_3_ and [Au_9_Ag_8_Cu_4_(SAdm)_4_(Dppm)_6_Cl_6_](SbF_6_)_3_ and **(C, D)** the ESI-MS spectra after reacting 3 h. For the top spectra of Figure 4D, the peaks with +3 charge indicate the [Au_9_Ag_8_Cu_4_(SAdm)_4_(Dppm)_6_Cl_6_]^3+^, [Au_9_Ag_9_Cu_3_(SAdm)_4_(Dppm)_6_Cl_6_]^3+^, [Au_9_Ag_10_Cu_2_(SAdm)_4_(Dppm)_6_Cl_6_]^3+^ and [Au_9_Ag_11_Cu_1_(SAdm)_4_(Dppm)_6_Cl_6_]^3+^. And For the bottom spectra of Figure 4D, the peaks with +2 charge indicate the {[Au_9_Ag_8_Cu_4_(SAdm)_4_(Dppm)_6_Cl_6_]+(SbF_6_)}^2+^, {[Au_9_Ag_9_Cu_3_(SAdm)_4_(Dppm)_6_Cl_6_]+(SbF_6_)}^2+^, {[Au_9_Ag_10_Cu_2_(SAdm)_4_(Dppm)_6_Cl_6_]+(SbF_6_)}^2+^ and {[Au_9_Ag_11_Cu_1_(SAdm)_4_(Dppm)_6_Cl_6_]+(SbF_6_)}^2+^.

Intercluster reactions between Au_9_Ag_12_ and Au_9_Ag_8_Cu_4_ (Abs._671nm_ = 0.3 for Au_9_Ag_12_ and Abs._712nm_ = 0.3 for Au_9_Ag_8_Cu_4_, respectively) are performed (Zhang et al., 2016; Khatun et al., 2020; Neumaier at al., 2021). As shown in Figure 7, the reaction was completed quickly (1 min), similar to the UV-vis spectrum that prolongs the reaction for 3 h. As shown in the Figures 7A,B, intercluster reactions produce a spectrum with 428, 482 and 702 nm, respectively. Learned from the Figures 7C,D, the products are Au_9_Ag_8_Cu_4_, Au_9_Ag_9_Cu_3_, Au_9_Ag_10_Cu_2_, Au_9_Ag_11_Cu_1_,respectively. Theoretical and experimental isotopic distributions of them matched perfectly as shown in Supplementary Figures S6, 7. This indicates the copper migration between Au_9_Ag_12_ and Au_9_Ag_8_Cu_4_ upon mixing in solution, similar to silver migration between Au_38_(SC_2_H_4_Ph)_24_ and doped Ag_x_Au_38-x_(SC_2_H_4_Ph)_24_ nanoclusters (Zhang et al., 2016).

## Conclusions

In conclusion, the regulation of surface structure of [Au_9_Ag_12_(SAdm)_4_(Dppm)_6_Cl_6_](SbF_6_)_3_ nanocluster *via* alloying produced an trimetallic nanocluster formulated as [Au_9_Ag_8_Cu_4_(SAdm)_4_(Dppm)_6_Cl_6_](SbF_6_)_3_. X-ray crystallography identifies that the Cu dopants prioritily replace the position of the silver of peripheral structures DppmAg_2_Cl_2_(SR)_2_. This controlled target metal exchange method may be extendable to other sized nanoclusters capped by multiple-ligands. Meanwhile the regulation of surface structure affected the CD spectra, DPV spectra, and stability. The [Au_9_Ag_12_(SAdm)_4_(Dppm)_6_Cl_6_](SbF_6_)_3_ and [Au_9_Ag_8_Cu_4_(SAdm)_4_(Dppm)_6_Cl_6_](SbF_6_)_3_ contribute to understanding of the structure-optical property relationship deeply.

## Data Availability

The datasets presented in this study can be found in online repositories. The names of the repository/repositories and accession number(s) can be found in the article/Supplementary Material.

## References

[B1] AbdulHalim L. G. Kothalawala N. Sinatra L. Dass A. Bakr O. M. (2014). Neat and Complete: Thiolate-Ligand Exchange on a Silver Molecular Nanoparticle. J. Am. Chem. Soc. 136 (45), 15865–15868. 10.1021/ja508860b 25345688

[B2] Barrabés N. Zhang B. Bürgi T. (2014). Racemization of Chiral Pd2Au36(SC2H4Ph)24: Doping Increases the Flexibility of the Cluster Surface. J. Am. Chem. Soc. 136 (41), 14361–14364. 10.1021/ja507189v 25251045

[B3] Bootharaju M. S. Joshi C. P. Parida M. R. Mohammed O. F. Bakr O. M. (2016). Templated Atom-Precise Galvanic Synthesis and Structure Elucidation of a [Ag24Au(SR)18]−Nanocluster. Angew. Chem. Int. Ed. 55 (3), 922–926. 10.1002/anie.201509381 26611172

[B4] Chai J. Yang S. Lv Y. Chong H. Yu H. Zhu M. Z. (2019). Exposing the Delocalized Cu−S π Bonds on the Au 24 Cu 6 (SPh T Bu) 22 Nanocluster and its Application in Ring‐Opening Reactions. Angew. Chem. Int. Ed. 58 (44), 15671–15674. 10.1002/anie.201907609 31437333

[B5] Chakraborty I. Pradeep T. (2017). Atomically Precise Clusters of Noble Metals: Emerging Link between Atoms and Nanoparticles. Chem. Rev. 117(12), 8208–8271. 10.1021/acs.chemrev.6b00769 28586213

[B6] Dias M. R. S. Leite M. S. (2019). Alloying: A Platform for Metallic Materials with On-Demand Optical Response. Acc. Chem. Res. 52 (10), 2881–2891. 10.1021/acs.accounts.9b00153 31305980

[B7] Ghosh A. Mohammed O. F. Bakr O. M. (2018). Atomic-Level Doping of Metal Clusters. Acc. Chem. Res. 51(12), 3094–3103. 10.1021/acs.accounts.8b00412 30452229

[B8] Hossain S. Ono T. Yoshioka M. Hu G. Hosoi M. Chen Z. (20182018). Thiolate-Protected Trimetallic Au∼20Ag∼4Pd and Au∼20Ag∼4Pt Alloy Clusters with Controlled Chemical Composition and Metal Positions. J. Phys. Chem. Lett. 9 (10), 2590–2594. 10.1021/acs.jpclett.8b00910 29709190

[B9] Jin R. Li G. Sharma S. Li Y. Du X. (2021). Toward Active-Site Tailoring in Heterogeneous Catalysis by Atomically Precise Metal Nanoclusters with Crystallographic Structures. Chem. Rev. 121(2), 567–648. 10.1021/acs.chemrev.0c00495 32941029

[B10] Jin R. Zeng C. Zhou M. Chen Y. (2016). Atomically Precise Colloidal Metal Nanoclusters and Nanoparticles: Fundamentals and Opportunities. Chem. Rev. 116, 10346–10413. 10.1021/acs.chemrev.5b00703 27585252

[B11] Jin S. Xu F. Du W. Kang X. Chen S. Zhang J. (2018a). Isomerism in Au-Ag Alloy Nanoclusters: Structure Determination and Enantioseparation of [Au9Ag12(SR)4(dppm)6X6]3+. Inorg. Chem. 57(9), 5114–5119. 10.1021/acs.inorgchem.8b00183 29624376

[B12] Jin S. Zou X. Xiong L. Du W. Wang S. Pei Y. (2018b). Bonding of Two 8‐Electron Superatom Clusters. Angew. Chem. Int. Ed. 57(51), 16768–16772. 10.1002/anie.201810718 30351512

[B13] Jin Y. Zhang C. Dong X.-Y. Zang S.-Q. Mak T. C. W. (2021). Shell Engineering to Achieve Modification and Assembly of Atomically-Precise Silver Clusters. Chem. Soc. Rev. 50(4), 2297–2319. 10.1039/D0CS01393E 33443527

[B14] Kang X. Abroshan H. Wang S. Zhu M. (2019a). Free Valence Electron Centralization Strategy for Preparing Ultrastable Nanoclusters and Their Catalytic Application. Inorg. Chem. 58(16), 11000–11009. 10.1021/acs.inorgchem.9b01545 31386346

[B15] Kang X. Wei X. Jin S. Yuan Q. Luan X. Pei Y. (2019b). Rational Construction of a Library of M29 Nanoclusters from Monometallic to Tetrametallic. Proc. Natl. Acad. Sci. USA 116(38), 18834–18840. 10.1073/pnas.1912719116 31488725 PMC6754594

[B16] Kang X. Xiong L. Wang S. Yu H. Jin S. Song Y. (2016). Shape-Controlled Synthesis of Trimetallic Nanoclusters: Structure Elucidation and Properties Investigation. Chem. Eur. J. 22(48), 17145–17150. 10.1002/chem.201603893 27754605

[B17] Khatun E. Chakraborty P. Jacob B. R. Paramasivam G. Bodiuzzaman M. Dar W. A. (2020). Intercluster Reactions Resulting in Silver-Rich Trimetallic Nanoclusters. Chem. Mater. 32(1), 611–619. 10.1021/acs.chemmater.9b04530

[B18] Khatun E. Ghosh A. Chakraborty P. Singh P. Bodiuzzaman M. Ganesan P. (2018). A Thirty-fold Photoluminescence Enhancement Induced by Secondary Ligands in Monolayer Protected Silver Clusters. Nanoscale 10(42), 20033–20042. 10.1039/C8NR05989F 30351319

[B19] Kwak K. Choi W. Tang Q. Kim M. Lee Y. Jiang D.-E. (2017). A Molecule-like PtAu24(SC6H13)18 Nanocluster as an Electrocatalyst for Hydrogen Production. Nat. Commun. 8(1), 14723. 10.1038/ncomms14723 28281526 PMC5353570

[B20] Li J. Li H. Yu H. Chai J. Li Q. Song Y. (2020). A Novel Geometric Structure of a Nanocluster with an Irregular Kernel: Ag30Cu14(TPP)4(SR)28. Dalton Trans. 49(23), 7684–7687. 10.1039/D0DT01142H 32510094

[B21] Neumaier M. Baksi A. Weis P. Schneider E. K. Chakraborty P. Hahn H. (2021). Kinetics of Intercluster Reactions between Atomically Precise Noble Metal Clusters [Ag25(DMBT)18]− and [Au25(PET)18]− in Room Temperature Solutions. J. Am. Chem. Soc. 143(18), 6969–6980. 10.1021/jacs.1c01140 33913724

[B22] Sharma S. Yamazoe S. Ono T. Kurashige W. Niihori Y. Nobusada K. (2016). Tuning the Electronic Structure of Thiolate-Protected 25-atom Clusters by Co-substitution with Metals Having Different Preferential Sites. Dalton Trans. 45(45), 18064–18068. 10.1039/c6dt03214a 27845455

[B23] Sun S. Liu H. Xin Q. Chen K. Chen K. Liu S. H. (2021). Atomic Engineering of Clusterzyme for Relieving Acute Neuroinflammation through Lattice Expansion. Nano Lett. 21(6), 2562–2571. 10.1021/acs.nanolett.0c05148 33720739

[B24] Wang S. Li Q. Kang X. Zhu M. (2018). Customizing the Structure, Composition, and Properties of Alloy Nanoclusters by Metal Exchange. Acc. Chem. Res. 51(11), 2784–2792. 10.1021/acs.accounts.8b00327 30387990

[B25] Xu S. Li W. Zhao X. Wu T. Cui Y. Fan X. (2019). Ultrahighly Efficient and Stable Fluorescent Gold Nanoclusters Coated with Screened Peptides of Unique Sequences for Effective Protein and Serum Discrimination. Anal. Chem. 91(21), 13947–13952. 10.1021/acs.analchem.9b03463 31558029

[B26] Yan J. Su H. Yang H. Hu C. Malola S. Lin S. (2016). Asymmetric Synthesis of Chiral Bimetallic [Ag28Cu12(SR)24]4- Nanoclusters via Ion Pairing. J. Am. Chem. Soc. 138(39), 12751–12754. 10.1021/jacs.6b08100 27626935

[B27] Yan J. Teo B. K. Zheng N. (2018). Surface Chemistry of Atomically Precise Coinage-Metal Nanoclusters: From Structural Control to Surface Reactivity and Catalysis. Acc. Chem. Res. 51(12), 3084–3093. 10.1021/acs.accounts.8b00371 30433756

[B28] Yan N. Liao L. Yuan J. Lin Y.-j. Weng L.-H. Yang J. (2016). Bimetal Doping in Nanoclusters: Synergistic or Counteractive? Chem. Mater. 28(22), 8240–8247. 10.1021/acs.chemmater.6b03132

[B29] Yang H. Wang Y. Lei J. Shi L. Wu X. Mäkinen V. (2013). Ligand-Stabilized Au13Cux (X = 2, 4, 8) Bimetallic Nanoclusters: Ligand Engineering to Control the Exposure of Metal Sites. J. Am. Chem. Soc. 135(26), 9568–9571. 10.1021/ja402249s 23789787

[B30] Yang S. Chai J. Chen T. Rao B. Pan Y. Yu H. (2017). Crystal Structures of Two New Gold-Copper Bimetallic Nanoclusters: CuxAu25-x(PPh3)10(PhC2H4S)5Cl22+ and Cu3Au34(PPh3)13(tBuPhCH2S)6S23+. Inorg. Chem. 56(4), 1771–1774. 10.1021/acs.inorgchem.6b02016 28140578

[B31] Yao Q. Chen T. Yuan X. Xie J. (2018). Toward Total Synthesis of Thiolate-Protected Metal Nanoclusters. Acc. Chem. Res. 51(6), 1338–1348. 10.1021/acs.accounts.8b00065 29792422

[B32] Zeng C. Jin R. (2017). Chiral Gold Nanoclusters: Atomic Level Origins of Chirality. Chem. Asian J. 12(15), 1839–1850. 10.1002/asia.201700023 28653468

[B33] Zhang B. Salassa G. Bürgi T. (2016). Silver Migration between Au38(SC2H4Ph)24 and Doped AgxAu38−x(SC2H4Ph)24 Nanoclusters. Chem. Commun. 52(59), 9205–9207. 10.1039/c6cc04469g 27352728

[B34] Zheng Y. Wu J. Jiang H. Wang X. (2021). Gold Nanoclusters for Theranostic Applications. Coord. Chem. Rev. 431, 213689. 10.1016/j.ccr.2020.213689

[B35] Zou X. Li Y. Jin S. Kang X. Wei X. Wang S. (2020). Doping Copper Atoms into the Nanocluster Kernel: Total Structure Determination of [Cu30Ag61(SAdm)38S3](BPh4). J. Phys. Chem. Lett. 11(6), 2272–2276. 10.1021/acs.jpclett.0c00271 32141753

