# Supplementary material for: Regulation of Surface Structure of [Au9Ag12(SAdm)4(Dppm)6Cl6](SbF6)3 Nanocluster via Alloying
Source: Front Chem. 2022 Jan 24;9:793339. doi: 10.3389/fchem.2021.793339 (PMC8819595; doi:10.3389/fchem.2021.793339)
Supplement: Supplementary file 1 [file DataSheet1.docx]

Supplementary Material

Regulation of Surface Structure of [Au_9_Ag_12_(SAdm)_4_(Dppm)_6_Cl_6_](SbF_6_)_3_  Nanoclusters via Alloying

**Huijuan Deng,ǂ Xiaowu Li,ǂ Xiaoxun Yan, Shan Jin, * and Manzhou Zhu***

Department of Chemistry and Centre for Atomic Engineering of Advanced Materials, Key Laboratory of Structure and Functional Regulation of Hybrid Materials of Ministry of Education, Institutes of Physical Science and Information Technology and Anhui Province Key Laboratory of Chemistry for Inorganic/Organic Hybrid Functionalized Materials, Anhui University, Hefei, Anhui 230601, China.

*** Correspondence:**

Shan Jin-Email: jinshan@ahu.edu.cn

Manzhou Zhu - Email: [zmz@ahu.edu.cn](mailto:zmz@ahu.edu.cn)


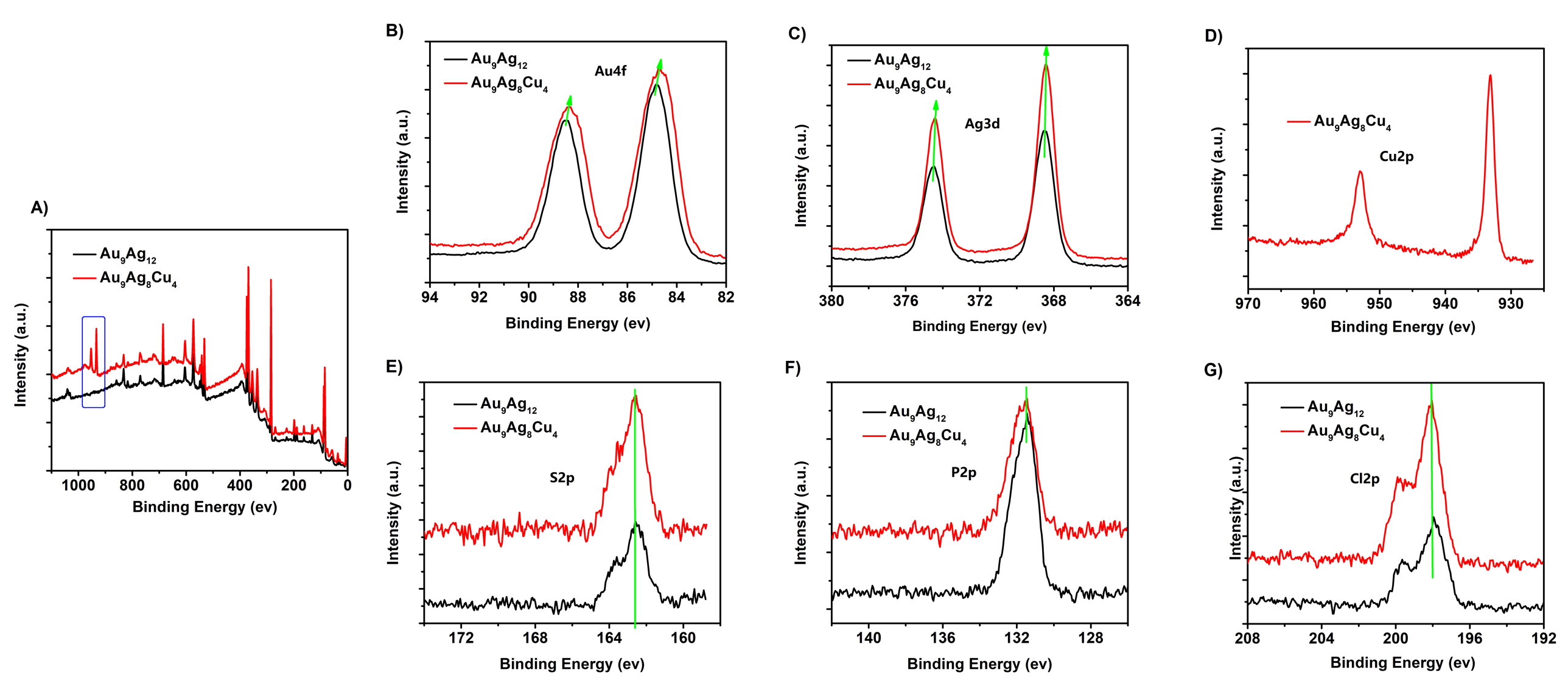
**Supplementary Figure 1.** X-ray photoelectron spectroscopy (XPS) of Au_9_Ag_12_ and Au_9_Ag_8_Cu_4_ nanoclusters. A) total spectra of XPS, B) Au4f, C) Ag3d, D) Cu2p, E) S2p, F) P2p and H) Cl2p.


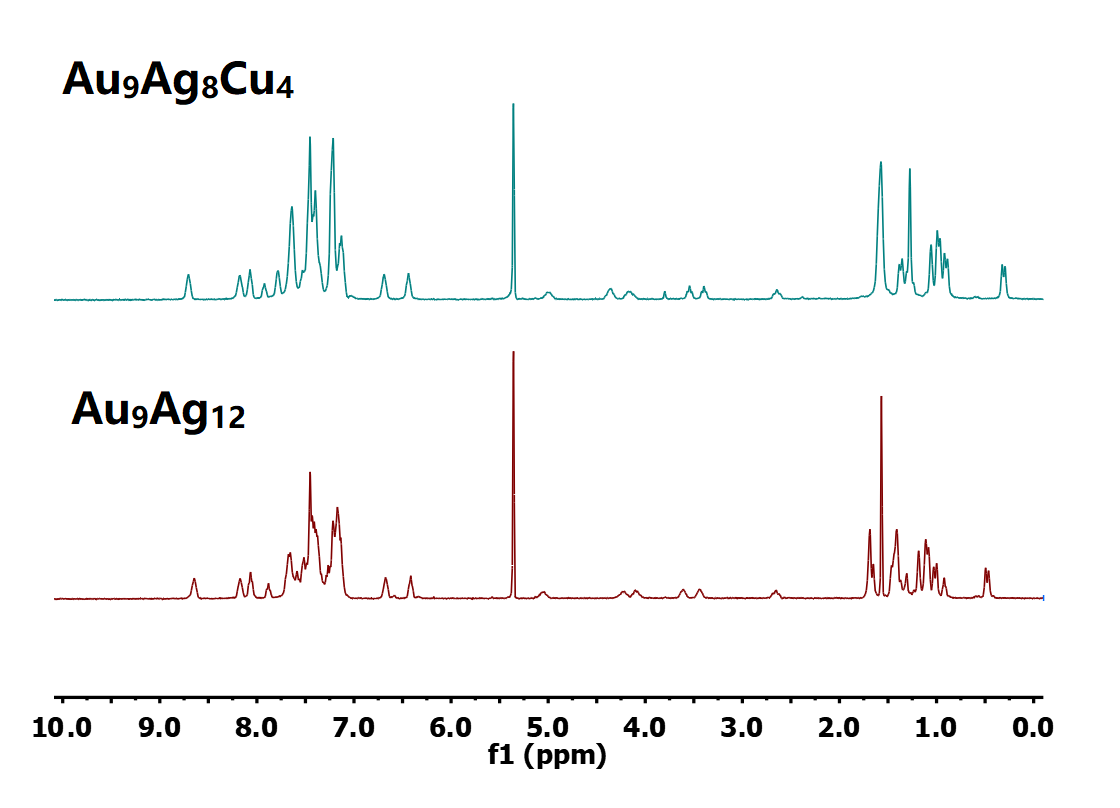


**Supplementary Figure 2.** H-NMR of Au_9_Ag_12_ and Au_9_Ag_8_Cu_4_ nanoclusters dissolved in CD_2_Cl_2_ solvent.


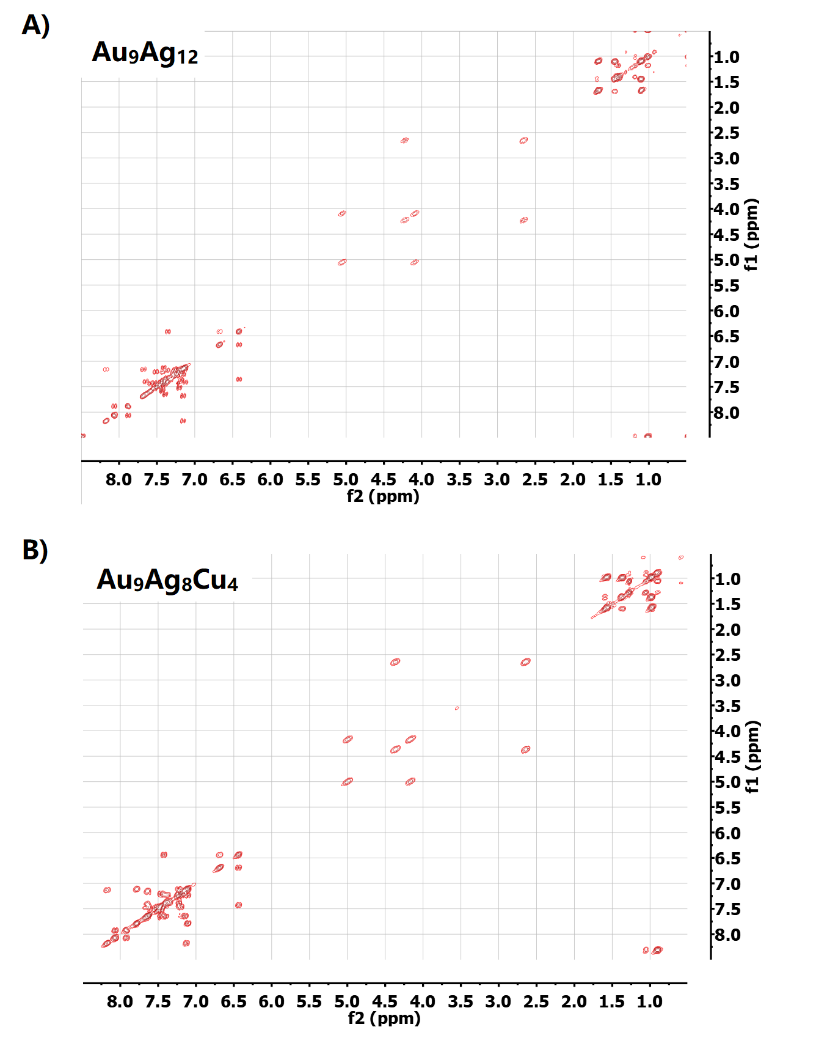


**Supplementary Figure 3.** ^1^H-^1^H COSY spectra of Au_9_Ag_12_ and Au_9_Ag_8_Cu_4_ nanoclusters dissolved in CD_2_Cl_2_ solvent.


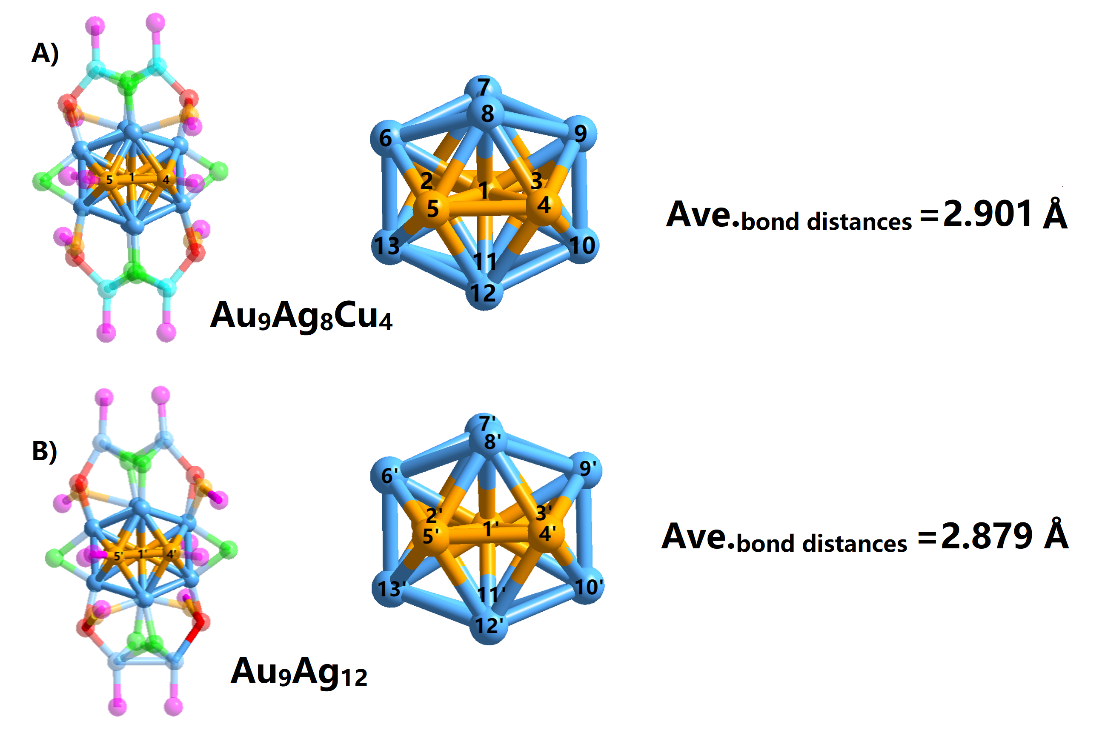


**Supplementary Figure 4.** The comparison of icosahedral Au_5_Ag_8_ in two nanoclusters.


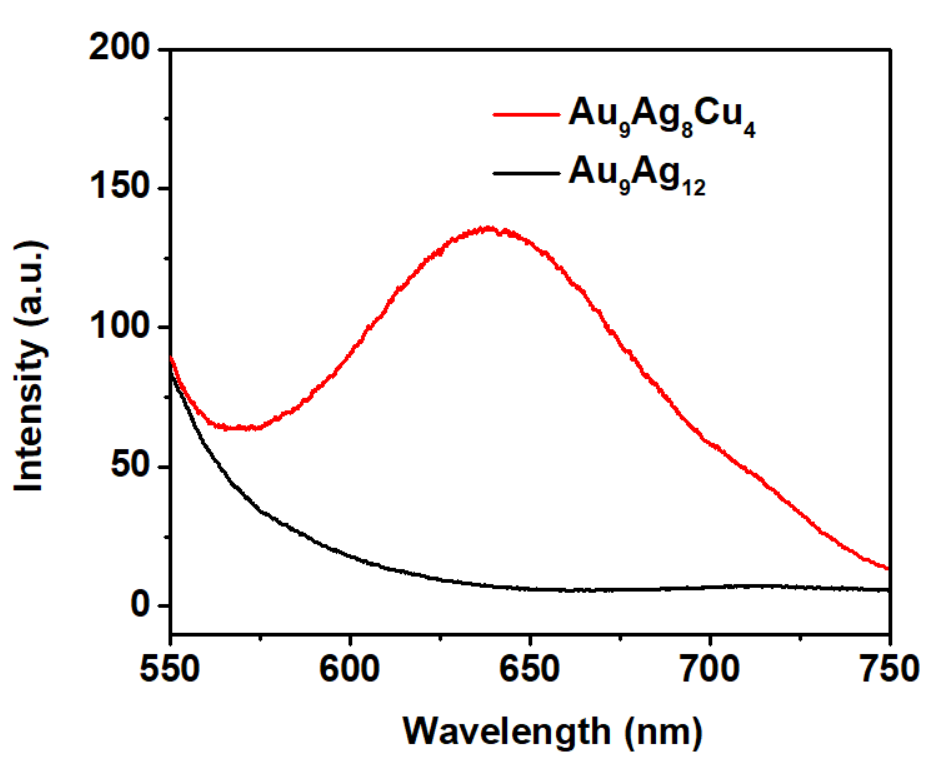


Supplementary Figure 5. The fluorescence spectra of Au_9_Ag_12_ (black line) and Au_9_Ag_8_Cu_4_ (red line) in CH_2_Cl_2_ solution (Ex.= 470 nm).


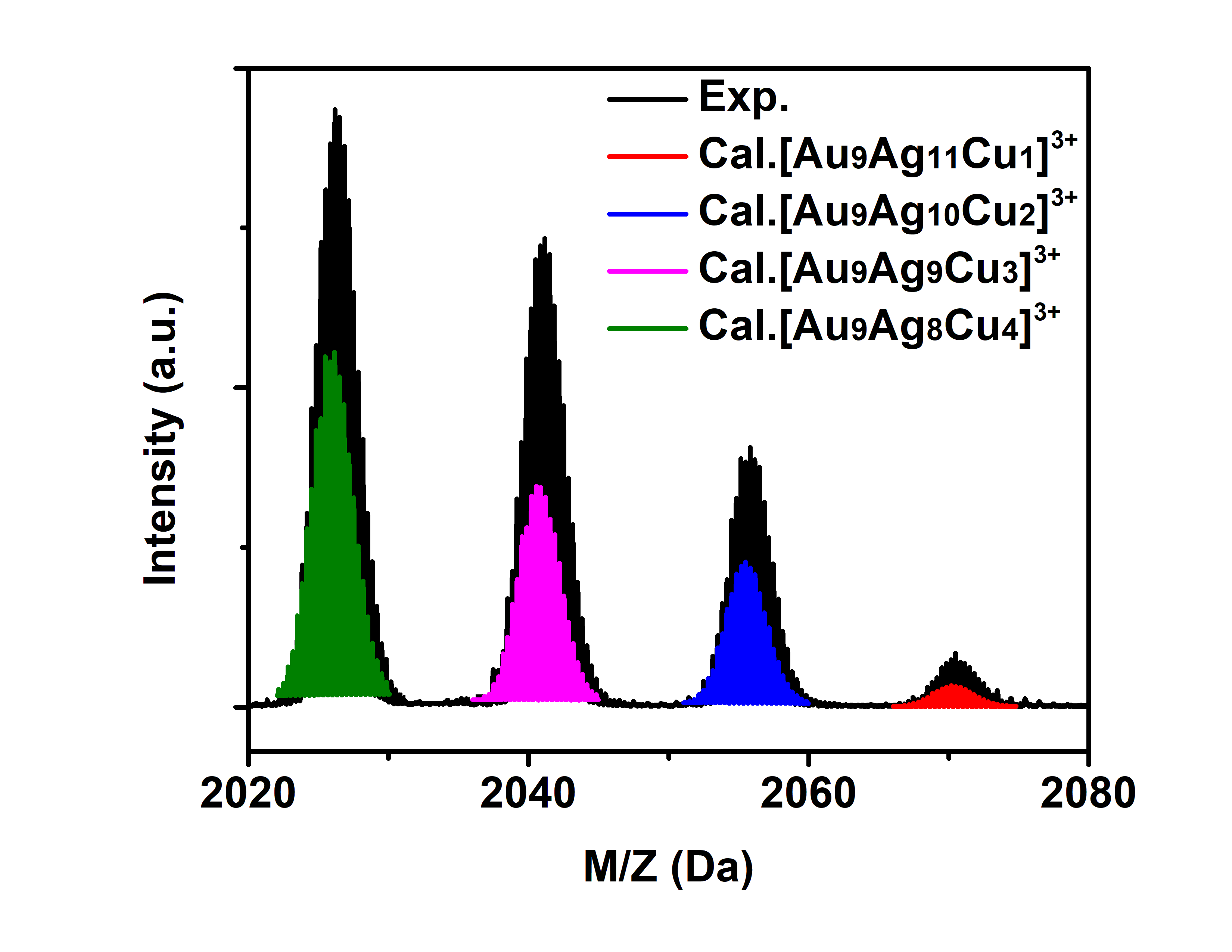


**Supplementary Figure 6.** Electrospray ionization mass (ESI-MS) spectra of the product with the characteristic peak separation of m/z 0.33 Da after intercluster reactions between Au_9_Ag_12_ and Au_9_Ag_8_Cu_4_.


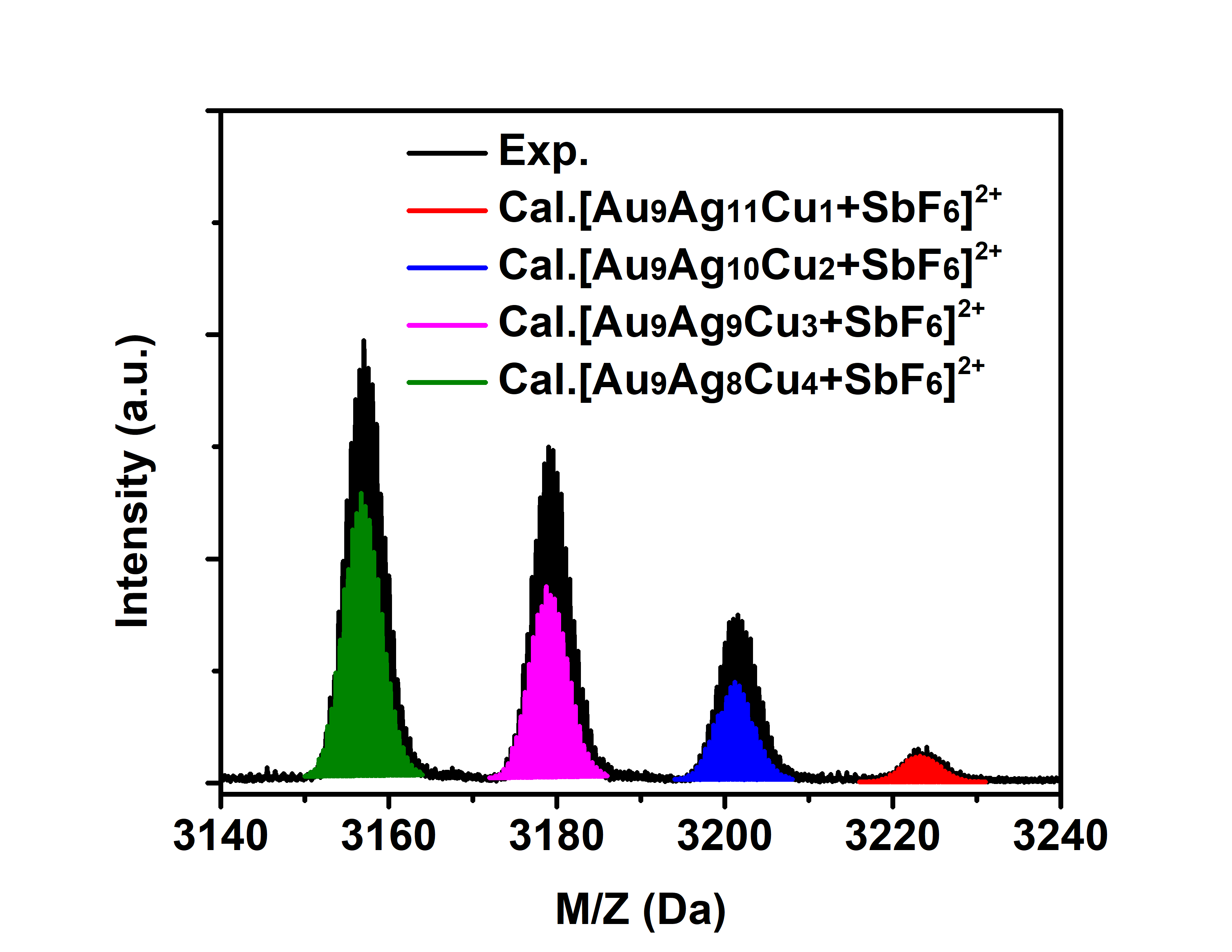


**Supplementary Figure 7.** Electrospray ionization mass (ESI-MS) spectra of the product with the characteristic peak separation of m/z 0.5 Da after intercluster reactions between Au_9_Ag_12_ and Au_9_Ag_8_Cu_4_

**Table S1. Crystal data and structure refinement for AuAgCu Alloy.**

| Empirical formula | C_190_H_192_Ag_8_Au_9_Cl_6_Cu_4_F_18_P_12_S_4_Sb_3_ |
| --- | --- |
| Formula weight | 6785.07 |
| Temperature/K | 130(2) |
| Crystal system | monoclinic |
| Space group | P2_1_/n |
| a/Å | 26.0358(18) |
| b/Å | 32.5111(12) |
| c/Å | 29.0574(12) |
| α/° | 90 |
| β/° | 103.055(3) |
| γ/° | 90 |
| Volume/Å^3^ | 23960(2) |
| Z | 4 |
| Radiation | CuKα (λ = 1.54178) |
| Independent reflections | 43830 [R_int_ = 0.0506, R_sigma_ = 0.0421] |
| Final R indexes [I>=2σ (I)] | R_1_ = 0.0341, wR_2_ = 0.0779 |
| Final R indexes [all data] | R_1_ = 0.0522, wR_2_ = 0.0852 |
| Largest diff. peak/hole / e Å^-3^ | 2.40/-1.82 |

**Table S2 Crystal data and structure refinement for AuAg Alloy.**

| Empirical formula | C_191_H_194.5_Ag_12_Au_9_Cl_8_F_18_P_12_S_4_Sb_3_ |
| --- | --- |
| Formula weight | 7047.82 |
| Temperature/K | 296.15 |
| Crystal system | monoclinic |
| Space group | P2_1_/c |
| a/Å | 19.2119(6) |
| b/Å | 29.7928(10) |
| c/Å | 39.6756(13) |
| α/° | 90 |
| β/° | 99.672(2) |
| γ/° | 90 |
| Volume/Å^3^ | 22386.6(13) |
| Z | 4 |
| Radiation | MoKα (λ = 0.71073) |
| Independent reflections | 51296 [R_int_ = 0.1422, R_sigma_ = 0.1818] |
| Final R indexes [I>=2σ (I)] | R_1_ = 0.0968, wR_2_ = 0.2494 |
| Final R indexes [all data] | R_1_ = 0.2013, wR_2_ = 0.3103 |
| Largest diff. peak/hole / e Å^-3^ | 6.86/-6.36 |
